# Genes Involved in Degradation of *para*-Nitrophenol Are Differentially Arranged in Form of Non-Contiguous Gene Clusters in *Burkholderia* sp. strain SJ98

**DOI:** 10.1371/journal.pone.0084766

**Published:** 2013-12-23

**Authors:** Surendra Vikram, Janmejay Pandey, Shailesh Kumar, Gajendra Pal Singh Raghava

**Affiliations:** 1 Bioinformatics Center, CSIR-Institute of Microbial Technology, Chandigarh, India; 2 Microbial Type Culture Collection Center, CSIR-Institute of Microbial Technology, Chandigarh, India; Aligarh Muslim University, India

## Abstract

Biodegradation of *para*-Nitrophenol (PNP) proceeds via two distinct pathways, having 1,2,3-benzenetriol (BT) and hydroquinone (HQ) as their respective terminal aromatic intermediates. Genes involved in these pathways have already been studied in different PNP degrading bacteria. *Burkholderia* sp. strain SJ98 degrades PNP via both the pathways. Earlier, we have sequenced and analyzed a ~41 kb fragment from the genomic library of strain SJ98. This DNA fragment was found to harbor all the lower pathway genes; however, genes responsible for the initial transformation of PNP could not be identified within this fragment. Now, we have sequenced and annotated the whole genome of strain SJ98 and found two ORFs (viz., *pnpA* and *pnpB*) showing maximum identity at amino acid level with *p*-nitrophenol 4-monooxygenase (PnpM) and p-benzoquinone reductase (BqR). Unlike the other PNP gene clusters reported earlier in different bacteria, these two ORFs in SJ98 genome are physically separated from the other genes of PNP degradation pathway. In order to ascertain the identity of ORFs *pnpA* and *pnpB*, we have performed *in-vitro* assays using recombinant proteins heterologously expressed and purified to homogeneity. Purified PnpA was found to be a functional PnpM and transformed PNP into benzoquinone (BQ), while PnpB was found to be a functional BqR which catalyzed the transformation of BQ into hydroquinone (HQ). Noticeably, PnpM from strain SJ98 could also transform a number of PNP analogues. Based on the above observations, we propose that the genes for PNP degradation in strain SJ98 are arranged differentially in form of non-contiguous gene clusters. This is the first report for such arrangement for gene clusters involved in PNP degradation. Therefore, we propose that PNP degradation in strain SJ98 could be an important model system for further studies on differential evolution of PNP degradation functions.

## Introduction

*para-*Nitrophenol (PNP) is a toxic and bio-refractory organic pollutant which releases into the environment via industrial waste and agricultural application of parathion and methyl parathion derived pesticides [1-4]. It has been categorized as a priority environmental pollutant and is reported to be hazardous to humans, and a number of animal models [5-7]. Several bacterial strains have been isolated and characterized for their ability to metabolize PNP as the sole source of carbon, nitrogen and energy [8-15]. Furthermore, two mutually independent oxidative pathways for microbial aerobic degradation of PNP have also been well elucidated in number of PNP degrading bacteria. The 1,2,4-benzenetriol (BT) pathway, commonly reported in Gram-positive bacteria, proceeds via transformation of PNP to BT [13,16]. On the other hand, hydroquinone (HQ) pathway, preferentially found in Gram-negative bacteria involves conversion of PNP to hydroquinone (HQ) via *p*-benzoquinone (BQ) [17]. A few studies have reported for cloning and characterization of gene(s) and/or gene cluster(s) involved in PNP degradation via BT pathway and HQ pathway [16,18-21]. Interestingly, only a very few studies have reported deviation from the above generalization with regards to distribution of PNP degradation pathways amongst Gram positive and Gram negative bacterial isolates. Recently, Zhang et al., (2012) have shown involvement of both 4-Nitrocatechol (NC)/BT and HQ pathways for degradation of PNP in *Pseudomonas* sp. 1-7 [22]. In a concurrent report, we have also shown the involvement of both BT and HQ pathways in the degradation of PNP by *Burkholderia* sp. strain SJ98 [23]. The biochemical analysis of samples collected during growth and resting cell studies with strain SJ98 on PNP showed the presence of characteristic intermediates of BT and HQ pathways. Additionally, annotation and genetic characterization of ~41 kb DNA fragment of a cosmid clone screened from the genomic library of strain SJ98 for harboring the open reading frame (ORF) *pnpC* which encodes for benzenetriol dioxygenase (BtD) has shown the presence of 5 ORFs (i.e., *pnpE2, pnpE1, pnpF, pnpD* and *pnpC*). These ORFs were later identified for encoding small subunit of Hydroquinone dioxygenase (HqD-SS), large subunit of Hydroquinone dioxygenase (HqD-LS), 4-Hydroxymuconic semialdehyde dehydrogenase (4-HMSD), BtD and Maleylacetate reductase (MaR) respectively [21,23]. The first three enzymes are well established for catalyzing reactions involved in the degradation of PNP via HQ pathway [17,24-26], while the latter two are known to be involved in BT pathway [13,16,20]. Noticeably, this ~41 kb DNA fragment did not show the presence of any gene(s) corresponding to the initial reaction(s) of either of the above two pathways [23]. 

In the present study, we have identified two ORFs (*pnp*A and *pnp*B) after the whole genome sequencing and annotation of strain SJ98 [27]. These ORFs share maximum sequence identity at amino acid level with previously characterized *p*-nitrophenol 4-monooxygenase (PnpM) and p-benzoquinone reductase (BqR) respectively. Surprisingly, ORFs *pnpA* and *pnpB* in strain SJ98 are found to be physically separated and positioned very far from all other genes involved in PNP degradation (identified earlier to be located on ~41 kb genomic fragment). Biochemical characterization of purified PnpA ascertained its identity as PnpM with NADH/FAD dependent oxidoreductase activity. In *in-vitro* activity assays, it catalyzed monooxygenation of PNP. Purified PnpB transformed BQ to HQ in the presence of NAD(P)H/FMN and therefore, its identity was ascertained as a BqR. This is the first report for such type of genetic arrangement of PNP degrading genes in any bacteria. In this report, we also presented a comparison of PNP degradation gene cluster identified and characterized from strain SJ98 with PNP degradation gene clusters of other bacteria. It is proposed that this study will enhance the understanding of ‘genetic and functional diversity’ of PNP degradation in microorganisms. Further, based on these observation it can be proposed that this strain could be used as a model system for studying the ‘phylogenetic evolution’ of the genes involved in microbial degradation of PNP and potentially other related nitroaromatic compounds, e.g. chloro-nitrophenols.

## Materials and Methods

### Bacterial strains, plasmids, primers, media and culture conditions

Bacterial strains and plasmids used in the present study are listed in table S1 while primers are listed in table S2. Cultures of strain SJ98 were grown in minimal salt medium (MSM) supplemented with appropriate concentrations (100–300 µM) of nitroaromatic compounds/degradation intermediates. MSM was prepared according to the composition as described earlier by Pandey et al., (2012) [28]. Wild type strain SJ98 was maintained on nutrient rich media (nutrient agar, nutrient broth) prepared according to the manufacturer’s suggestion. *E. coli* BL21 AI, *E. coli* Top10 and recombinant *E. coli* strains were grown in Luria-Bertani medium. Filter sterilized antibiotic viz., ampicillin or kanamycin was added at a final concentration of 100 μg ml^-1^ or 50 μg ml^-1^ respectively, wherever required. Cultures of strain SJ98 were grown at 30°C with aeration (180-200 rpm) while *E. coli* strains were grown at different incubation temperatures (25°C, 30°C and 37°C) with aeration (200 rpm) as required. 

### Chemicals

The standards of *p*-nitrophenol (PNP), *m*-nitrophenol (MNP), o-nitrophenol (ONP), 4-nitrocatechol (4-NC), Benzoquinone (BQ), Hydroquinone (HQ), chlorohydroquninone (CHQ), 3-methyl-4-nitrophenol (3M4NP), 2-chloro-4-nitrophenol (2C4NP), 4-chloro-3-nitrophenol (4C3NP), 4-chloro-2-nitrophenol (4C2NP), 2,6-dichlorophenol (2,4-DCP) 2,4-dinitrophenol (2,4DNP) and 2,5-dinitrophenol (2,5-DNP), and all expected metabolites were obtained from Sigma Aldrich, (St, Louis, MO, USA). All other chemicals used in the study were of the purest grade available.

### Sequencing and analysis of strain SJ98 genome

Whole genome of *Burkholderia* sp. strain SJ98 was sequenced on Roche’s 454 and Illumina GAIIX platform; annotated by RNAmmer [29] and PGAAP pipeline. Annotated genome sequence is now available at National Center for Biotechnology Information (NCBI) GenBank database [27].

### Identification of *pnpA* and *pnpB*

The annotated genome sequence of strain SJ98 was searched and analyzed for the presence of ORFs with the putative function of PnpM and BqR. ORFs were identified using the ORF Finder program on the NCBI website (http://www.ncbi.nlm.nih.gov/projects/gorf). The deduced proteins were examined for sequence similarity with other proteins in the NCBI, GenBank database using BLASTN and BLASTP modules available from NCBI [30]. These analyses showed the presence of two physically associated ORFs (*pnpA* and *pnpB*) with maximum amino acid identity and putative functions of PnpM and BqR respectively. These ORFs were selected for further validation and characterization with heterologous expression, purification and biochemical studies.

### Comparison of PNP degrading gene clusters

Nucleotide sequences of previously characterized PNP degradation gene clusters were obtained from the GenBank (NCBI), and these sequences were saved into FASTA format. Analysis of ORFs orientation and comparison were done by Vector NTI v11.5.1 software (Invitrogen, life technologies, USA).

### Cloning and expression of *pnpA* and *pnpB*

DNA fragments corresponding to ORFs *pnpA* and *pnpB* were amplified from SJ98 genome using Hot-Star™ High-Fidelity Taq DNA Polymerase (Qiagen, GmBH, Germany) and primer pairs as listed in table S2. These primers were designed to incorporate *attB* sites corresponding to recombination site of the Gateway^TM^ entry vector (Invitrogen Inc. CA, USA) according to the manufacturer’s recommendations. The thermocycler program for amplifications was as follows: initial denaturation of genomic DNA at 95°C for 10 min followed by 29 cycles of denaturation at 95°C for 1 min, primer annealing at 55°C for 30 seconds and extension at 72°C for 2 min. Final extension was carried out at 72°C for 10 min. Resulting amplicons were analyzed with 1.0% agarose gel electrophoresis. Single amplicons with expected size for *pnpA* and *pnpB* were cloned in Gateway^TM^ entry vector pDONR221 using BP clonase enzyme (Invitrogen Inc. CA, USA) according to manufacturer’s recommendations. Sequences of the cloned amplicons were verified to ensure that no mutation has been introduced during PCR reaction. Sequencing of the amplicon was carried out with ABI-3130xl Genetic analyzer capillary sequencer (Applied Biosystems- Life Technologies, USA) using vector specific primers as shown in table S2. Subsequently, Gateway LR reactions were carried out to sub-clone *pnpA* and *pnpB* into the Gateway^TM^ N-terminal His-Tag expression vector viz., pDEST17. Recombinant plasmids obtained from the sub-cloning reactions were transformed into *E. coli* BL21-AI^TM^ (Invitrogen Inc. CA, USA) cell for protein expression according to the manufacturer’s instruction. Resulting transformants were designated as pDEST17-*pnpA* and pDEST17-*pnpB* and grown in Luria-Bertani broth at 37°C to an optical density (OD_600_) of ~0.5-0.6 and then induced for 12 hrs by adding L-Arabinose (0.2% w/v) at 25°C for expression of the target proteins. 

### Preparation of cell extract and protein purification

The cell pellets of induced recombinants were harvested from 250 ml culture by centrifugation, re-suspended into 8 ml of 50 mM potassium phosphate buffer, pH 8.0 containing imidazole (10 mM), NaCl (300 mM) and glycerol (10%) at pH 8.0 and lysed by ultrasonication. Over-expression of cloned genes was determined with SDS-PAGE analysis of lysed samples. Subsequently, cell free lysate with over-expressed PnpA and PnpB from 250 ml cultures were centrifuged at 13,000 x g for 20 min at 4°C to remove membrane fractions. Cell free lysate was further purified with affinity chromatography using 2 ml of Nickel-Nitrilotriacetic Acid (Ni^2+^ -NTA) Super-flow Cartridge (Qiagen, GmbH, Germany) according to supplier’s instructions. His-tagged PnpA and PnpB were separately allowed to bind to the resin equilibrated with 50 mM potassium phosphate buffer, pH 8.0, imidazole (10 mM), NaCl (300 mM) and glycerol (10%); washed twice with 10-15 ml of wash buffer containing 50 mM potassium phosphate buffer, pH 8.0 imidazole (30 mM), NaCl (300 mM) and glycerol (10%). His-tagged PnpA and PnpB were eluted in the buffer same as the binding buffer except that the final concentration of imidazole was 250 mM. Purified proteins were dialyzed twice in 50 mM potassium phosphate buffer, pH 8.0 containing NaCl (300 mM) and Glycerol (10%) at 4°C for 12-16 hrs. Protein concentrations were determined by Bradford reagent (Sigma, USA) using the standard concentration plot generated with known concentrations of Bovine Serum Albumin. Purification of the target protein was also scrutinized by SDS-PAGE analysis. Size exclusion chromatography of purified PnpA and PnpB was carried out using Shodex KW803 protein column on Agilent-1260 infinity series. Molecular mass and stoichiometry of the purified recombinant proteins was determined using linear standard curve generated with proteins of known molecular masses. Purified proteins were stored in storage buffer containing 50 mM potassium phosphate pH 8.0, NaCl (300 mM), Glycerol (10% v/v) at 4°C or -20°C. Aliquots of purified PnpA and PnpB proteins were frozen rapidly by plunging the sample vial in liquid nitrogen and then stored in -20°C. Later, PnpA and PnpB aliquots were thawed by incubating the sample vial under running tap water. 

### Enzyme activity of purified PnpA

The catalytic activity of purified PnpA was monitored with spectrophotometric enzyme assay specific for PnpM and performed according to the method described earlier by Spain et al., (1979) [31]. Briefly, the reaction mixtures consisted of 0.2 mM NADH, 0.02 mM flavin adenine dinucleotide (FAD), 10 µg of purified protein (PnpA), 100 µM substrate (PNP) in 50 mM phosphate buffer (pH 7.4) at a final reaction volume of 1.0 ml. The rationale for using a buffer with pH 7.4 for enzyme activity analysis of PnpA is explained later. Reaction was initiated with the addition of PNP at different concentrations ranging from 12.5- 250 µM. Reference cuvette contained all of the above components except the reaction substrate. The molar extinction coefficients of NADH and PNP were found to be maximum at 340 nm and 405 nm respectively. Therefore, positive activity of PnpA was determined by measuring the change (decrease) in spectral absorbance at above wavelengths corresponding to the consumption of NADH and PNP at regular interval. Kinetic properties of PnpA were determined by assays performed at different concentrations of PNP and NADH. Spectrophotometric analyses were performed on UV-visible spectrophotometer UV-1800 (Shimadzu Instrumentation, MD USA). Effects of temperature, pH and inhibitory metal ions on PnpA activity were studied with standard activity assay.

Alternatively, monooxygenase activity of PnpA was also studied with analytical chemistry approach, wherein 100 µg of purified His-6-PnpA was mixed with 100 µM of substrate (PNP) and 300 µM NADH, 50 µM FAD in 20 mM phosphate buffer (pH 7.4) in a total reaction volume of 10 ml and incubated at 25°C. Samples were collected and analysed for the quantitation of transformation of PNP into BQ by HPLC analysis according to the method described earlier by Ghosh et al., (2010) [32]. A brief description of the HPLC method used is described below. A positive PnpM activity was also measured by estimation of concentration of nitrite ion released in the reaction medium as determined by colorimetric methods described earlier [25,32]. Concentration of nitrite ion was estimated with reference of linear calibration curve generated with known concentrations (0.5 to 250 μM) of NaNO_2_. At least three time points from the linear parts of the nitrite release curves were used for the calculation of specific initial rates. 

### Substrate specificity of purified PnpA of strain SJ98

In order to evaluate the substrate specificity of PnpA, purified His-6-PnpA was subjected to HPLC analyses for NADH/FAD dependent monooxygenase activity using chemical analogues of PNP (viz., 4-NC, 2C4NP, 4-CP, 3,4-DCP, 4C3NP, 4C2NP, 2,4-DCP and 4-CC) as the reaction substrates. 100 µM of different substrates with 150 µM NADH and 50 µM FAD in 50 mM potassium phosphate buffer, pH 7.4 were incubated in a total reaction volume of 1 ml. Samples were analysed for the transformation of substrates and quantified using HPLC by comparing with known concentrations of authentic standards. 

### Enzyme activity of purified PnpB

For functional characterization of purified PnpB, spectrophotometric enzyme assay corresponding to BqR activities was performed according to the method described earlier [25,31]. Briefly, the reaction mixture for enzyme activity of His-6-PnpB contained 50 mM phosphate buffer, pH 7.2, 100 µM NADPH, 50 µM FMN and 15 µg of His-6-PnpB. The rationale for using a buffer with pH 7.2 is explained later. Positive BqR activity was determined by monitoring the transformation of BQ (molar extinction coefficient at 238.9 nm is 24,300 cm^-1^.M^-1^) to HQ according to the method described earlier [25,31,33]. The reaction was initiated by the addition of BQ to the reaction mixture. Reference cuvette contained each of the reaction components except the purified His-6-PnpB. Spectral analyses were carried out using Shimadzu UV-Visible spectrophotometer Model UV-1800 (Shimadzu Instrumentation, MD USA). Kinetic properties of PnpB were determined with enzyme assays performed at different concentrations of BQ and NAD(P)H. Activity assays were performed in triplicates, and arithmetic means were considered for estimation of the kinetic properties. 

### Analytical method

HPLC analyses of the samples collected from enzyme activity for PnpA on PNP and its chemical analogues were performed according to the method described earlier [32]. Briefly, samples were analyzed with a Waters 600 model equipped with Waters 996 photodiode array detector and C_18_ reversed-phase column (5 µm; 4.6 x 250 mm) (Waters Inc. MD, USA) operating at a column temperature of 25°C. Reaction substrate and product were separated with mobile phase consisting of 1% glacial acetic acid in methanol:1% glacial acetic acid in water with a gradient from 35:65 to 68:32 over 10 min at a flow rate of 1 ml.min^-1^.

### Phylogenetic analysis of PnpA and PnpB from different PNP degrading microorganisms

Phylogenetic analysis of PnpA and PnpB was carried out with amino acid sequences of PnpM and BqR previously characterized from different PNP degrading microorganisms. Amino- acid sequences were obtained from NCBI, GenBank database and subjected to multiple and pair-wise alignment using CLUSTALW module on the MEGA version 5 [34]. Phylogenetic and molecular evolutionary analyses were conducted using MEGA version 5. Phylogenetic trees were constructed with ‘Test Neighbor Joining’ approach using bootstrap values of 1000. Additionally, multiple sequence alignment of PnpB from *Burkholderia* sp. SJ98 (EKS70312.1) was performed with corresponding genes encoding BqR sequenced and reported from *Pseudomonas* sp. WBC-3 (ABU50909.1), *Pseudomonas putida* (ACN43576.1), *Pseudomonas* sp. 1-7 (ADB81393.1) and *Pseudomonas* sp. NyZ402 (ACZ51379.1) using ClustalX version 2.1 [35]. Further neighbor joining tree of this alignment was generated by using Jalview 2.8 [36].

### Nucleotide sequence accession number

This Whole Genome Shotgun project has been deposited at DDBJ/EMBL/GenBank under the accession AJHK00000000. The version described in this paper is the second version, AJHK02000000.2. Nucleotide sequences for ORFs *pnpA* and *pnpB* isolated and characterized from strain SJ98 have been deposited at GenBank under accession numbers BURK_019605 and BURK_019600 respectively. 

## Results and Discussion

### Identification of ORFs *pnpA* and *pnpB*

Genetic characterization of BT transforming gene cluster from strain SJ98 showed the presence of genes encoding cognate subunits of Hydroquinone dioxygenase (HqD) and 4-HMSD, BtD and MaR [23]. Noticeably, annotation of BT and HQ transforming gene cluster from strain SJ98 did not show the presence of either PnpM that would catalyze initial removal of nitro group as nitrite and formation of HQ or PNP-hydroxylase that would catalyze transformation of PNP into 4-NC and BT [21,23]. Furthermore, putative gene(s) with the above function could not be identified within a genomic DNA fragment extending ~41 Kb from the BtD gene [23].

**Figure 1:**
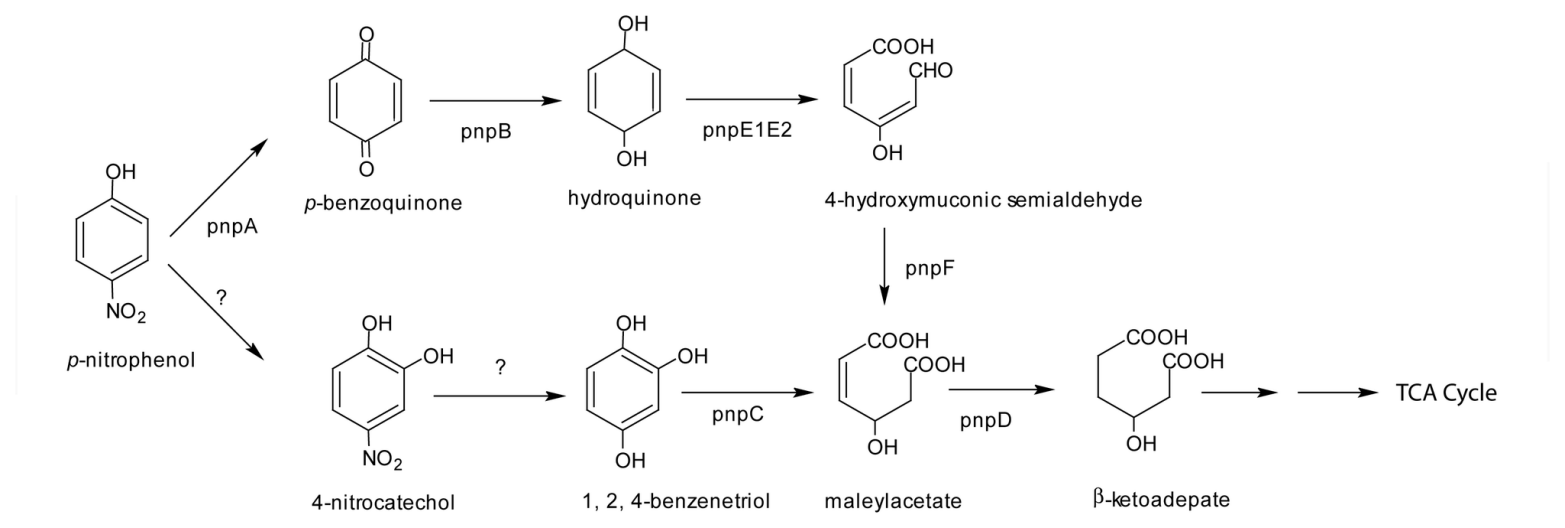
Predicted positions for the activity of *pnpA* and *pnpB* in *p*-nitrophenol (PNP) degradation pathway of strain SJ98.

Genome annotation of strain SJ98 showed the presence of an ORF (designated as *pnpA*) having conserved FAD and NADH binding motif- ‘GXXXLXGDAAH’ (where ‘X’ is any amino acid) as reported earlier [25] and maximum sequence similarity (78% identity at amino acid level for the ORF product) with PnpM of *Pseudomonas* sp. WBC-3 (accession no. ABU50908.1). Another ORF (designated as *pnpB*) has shown maximum sequence similarity (88% identity at amino acid level) with BqR of *Pseudomonas* sp. WBC-3 (accession no. ABU50909.1). Noticeably, the above ORFs were found to be physically separated from the other lower pathway genes of PNP degradation earlier identified from strain SJ98. Based on the sequence similarities, at this point of the study, we propose that the ORFs *pnpA* and *pnpB* could be potentially involved in PNP degradation by strain SJ98. The proposed position for proteins encoded by ORFs *pnpA* and *pnpB* on the degradation pathway of PNP is shown in Figure 1.

### Comparison of PNP gene cluster of strain SJ98 with those reported from other bacteria

**Figure 2:**
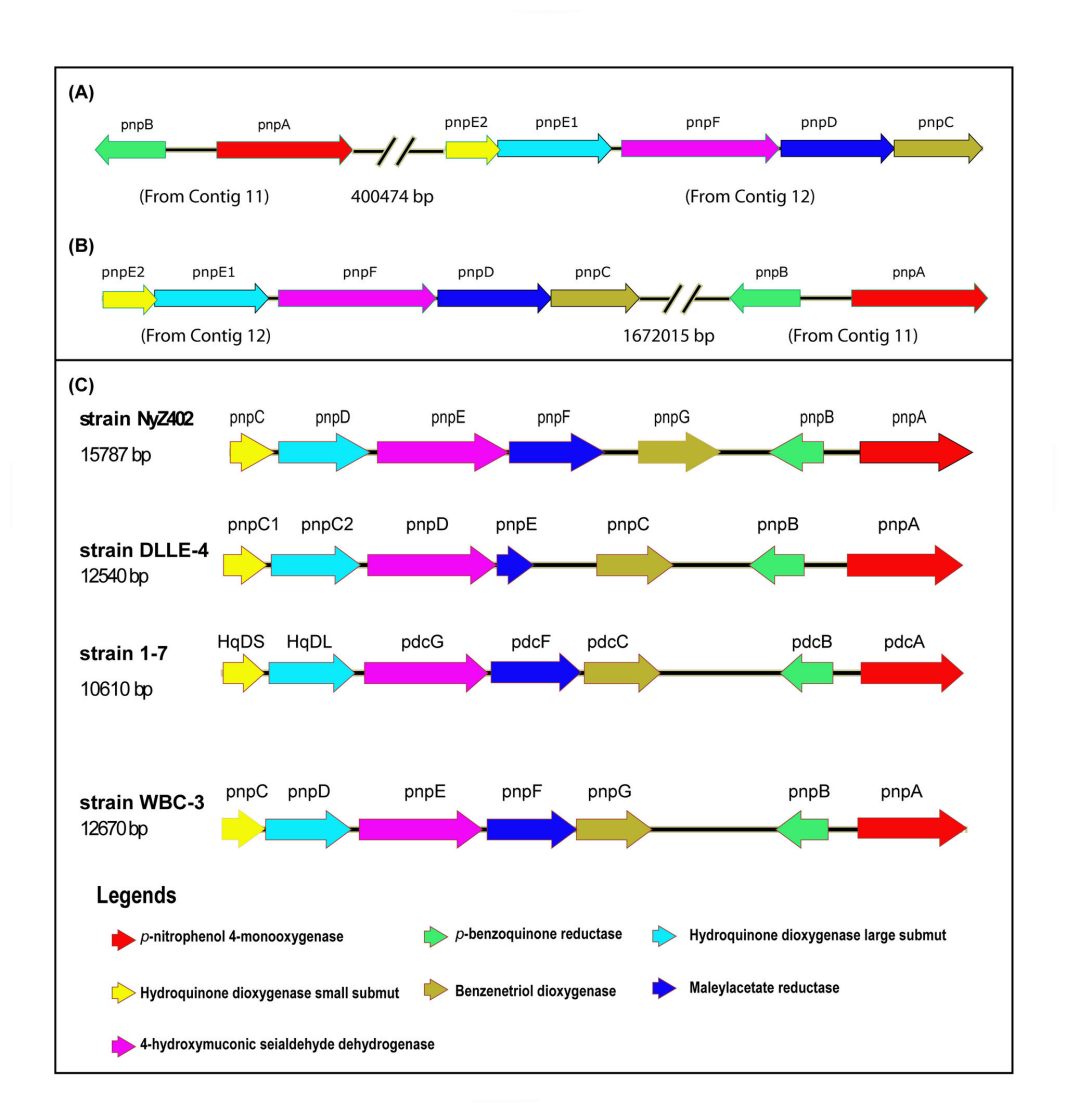
Comparison of *p*-nitrophenol (PNP) degradation gene clusters from different bacterial strains, schematic representation of the ORFs and distance between the gene clusters when arranged together as shown in *pnpAB* and *pnpCDE1E2F* (A) schematic representation of distance between Contig 11 (NZ_AJHK02000011.1) and contigs 12 (NZ_AJHK02000012.1) (B) if contigs 12 and then contigs 11 placed in series together then distance between the gene clusters and (C) PNP degradation gene clusters found in the other PNP degrading bacteria *Pseudomonas* sp. NyZ402 (GU123925.1), *Pseudomonas putida* DLLE-4 (FJ376608), *Pseudomonas* sp. 1-7 (FJ821777) and *Pseudomonas* sp. WBC-3 (EF577044). Similar genes have been assigned by the same colors.

It was observed from the whole genome sequence of strain SJ98 that the gene cluster *pnpAB* was present on contig 12 whereas gene cluster *pnpE1E2FCD* which harbored the lower pathway genes of PNP degradation was present on contig 11. Further attempts were made to find out the distance between these gene clusters in SJ98 genome. There are two possibilities for relative positions of contig 11 and 12: if both the contigs are joined back to back in the order of contig no. 11 followed by contig no. 12, then calculated distance between these gene clusters (*pnpAB* and *pnpE1E2FCD*) would be >1.6 Mb (Figure 2A), whereas if both the contigs are joined in the reverse order, then the distance between these two cluster would be >0.4 Mb (Figure 2B). Either way, this observation is different from the earlier reports pertaining to the organization of PNP degradation gene clusters from different PNP degrading organisms which showed the occurrence of all of the PNP degradation genes clustered together in only one gene cluster/operon [22,24,25,37] (Figure 2). 

Complete PNP gene clusters (which include the genes encoding the enzymes PnpM, BqR, HqD, 4-HMSD, BtD and MaR) previously reported from other microorganisms have been found to be present within DNA fragments ranging from ~9 to 15 kb [22,24,25,37]. Interestingly, the physical organization of the lower pathway genes (PNP degradation) in strain SJ98, Nyz402, DLL-E4, 1-7 and WBC3 is almost identical (Figure 2A, 2B and 2C). On the contrary, in strain SJ98, the PNP degradation genes are separated into two non-contiguous gene clusters viz., *pnpAB* and *pnpE1E2FCD* that are physically separated from one another (Figure 2). 

G+C content analysis of *pnpA* and *pnpB* genes found to be almost similar to the G+C content for the whole genome of strain SJ98 suggesting that these genes may not have been inherited by the horizontal gene transfer mechanism. Also at this point of our understanding, it would be premature to predict or comment upon the mechanism for such divergence. However, we propose that it would be very interesting to work out the molecular details of this observation. Similar separation of the metabolic operons has been reported in a number of earlier reports [38–40]. Zaslaver et al., (2006) have reported that the partitioning of given set of genes is not same in every organism [38]. Gene re-arrangement can take place in any organisms due to mutational event such as gene duplication, which forms new operons or genome rearrangement that can split the existing operon or lateral gene transfer from other organisms [38,39]. However, to the best of authors’ knowledge, a similar partitioning of gene clusters has not been reported with regards to the degradation of PNP. 

### Cloning, expression of ORFs *pnpA, pnpB* and purification of PnpA and PnpB

**Figure 3:**
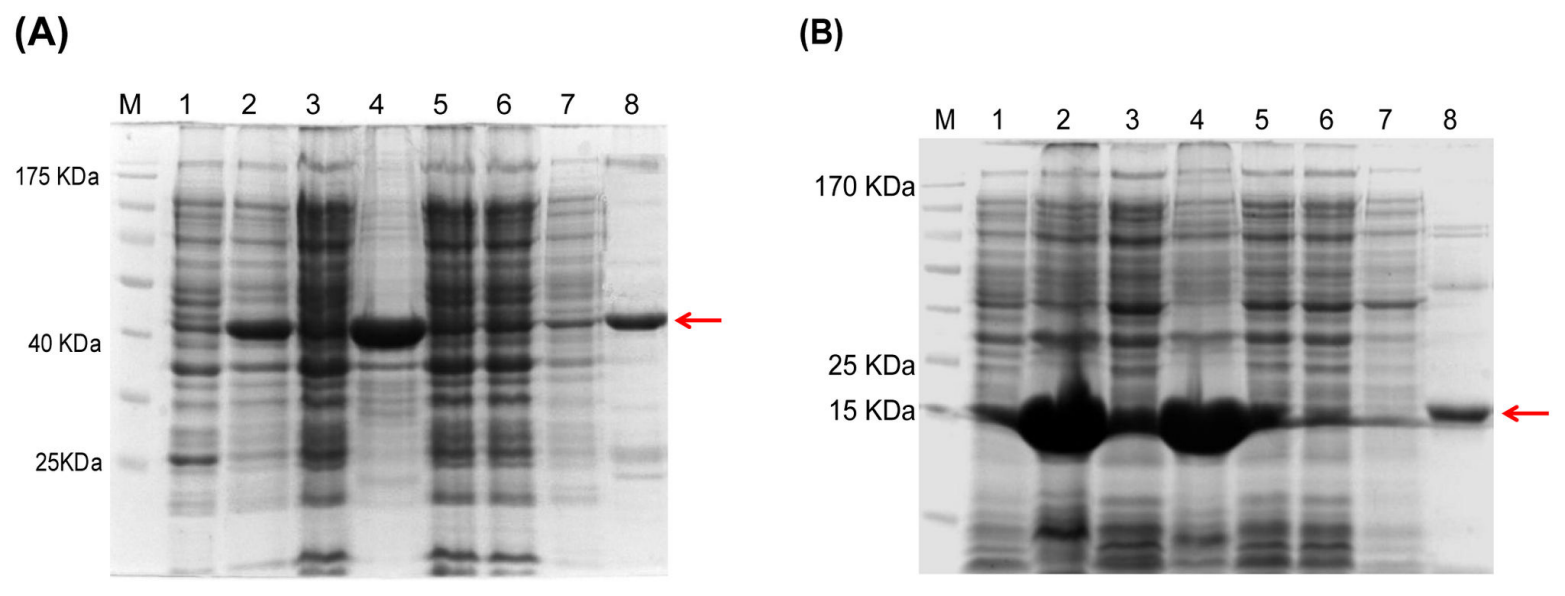
Representative SDS PAGE gel picture of (A) His-6-PnpA purification by Ni-NTA chromatography, Lane M) Marker, Lane 1) uninduced, 2) induced whole cell lysate, 3) Induced supernatant, 4) pellet, 5) & 6) flow through, 7) wash and lane 8) Elution of His-6-PnpA is showing with a red arrow.(**B**) His-6-PnpB purification by Ni-NTA chromatography, Lane M) Marker, Lane1) uninduced , 2) induced whole cell lysate, 3) Induced supernatant, 4) pellet, 5) & 6) flow through, 7) wash and lane 8) Elution of His-6-PnpB is showing with a red arrow.

ORF *pnp*A was cloned, expressed and subsequently His-6-PnpA was purified to homogeneity with a yield of ~5 mg of PnpA from 1 liter of culture. In SDS PAGE analysis, purified His-6-PnpA protein (>90%) was found to have an estimated molecular weight of ~46 kDa (Figure 3A). Gel filtration chromatography of functionally active His-6-PnpA indicated it to be a monomer. This observation is in agreement with the earlier report that has shown PnpM to be a monomer [25].

Similarly ORF *pnp*B was cloned, expressed and His-6-PnpB protein purified with an identical procedure as used for purification of His-6-PnpA protein to obtain a yield of ~12 mg of PnpB protein from 1 liter of culture. In SDS PAGE analysis, purified His-6-PnpB showed a single band of >90% purity at an apparent molecular mass of ~21 kDa (Figure 3B). Gel filtration chromatography of functionally active His-6-PnpB showed it to be a monomer (Figure S1A). The corresponding size exclusion standard curve generated with proteins of known molecular masses is shown in Figure S1B. Characteristically, majority of the earlier reports have shown functional BqR to be a NAD(P)H and FMN dependent dimer(s) and/or tetramers [25,41,42]. Result obtained during the present study with regards to the oligomeric state BqR of strain SJ98 represented one of the very few examples showing occurrence of a functional BqR as a monomeric protein. Lee et al., (2007) reported isolation and characterization of monomeric quinone reductase involved in the degradation of aromatic compound including lignin by white rot fungi viz., *Trametes versicolor* [43]. Subsequent biochemical characterization clearly established that despite being functional monomer, the BqR from strain SJ98 exhibited somewhat similar biochemical characteristics to those reported with the other BqRs. 

### Biochemical and kinetic properties of PnpA

**Figure 4:**
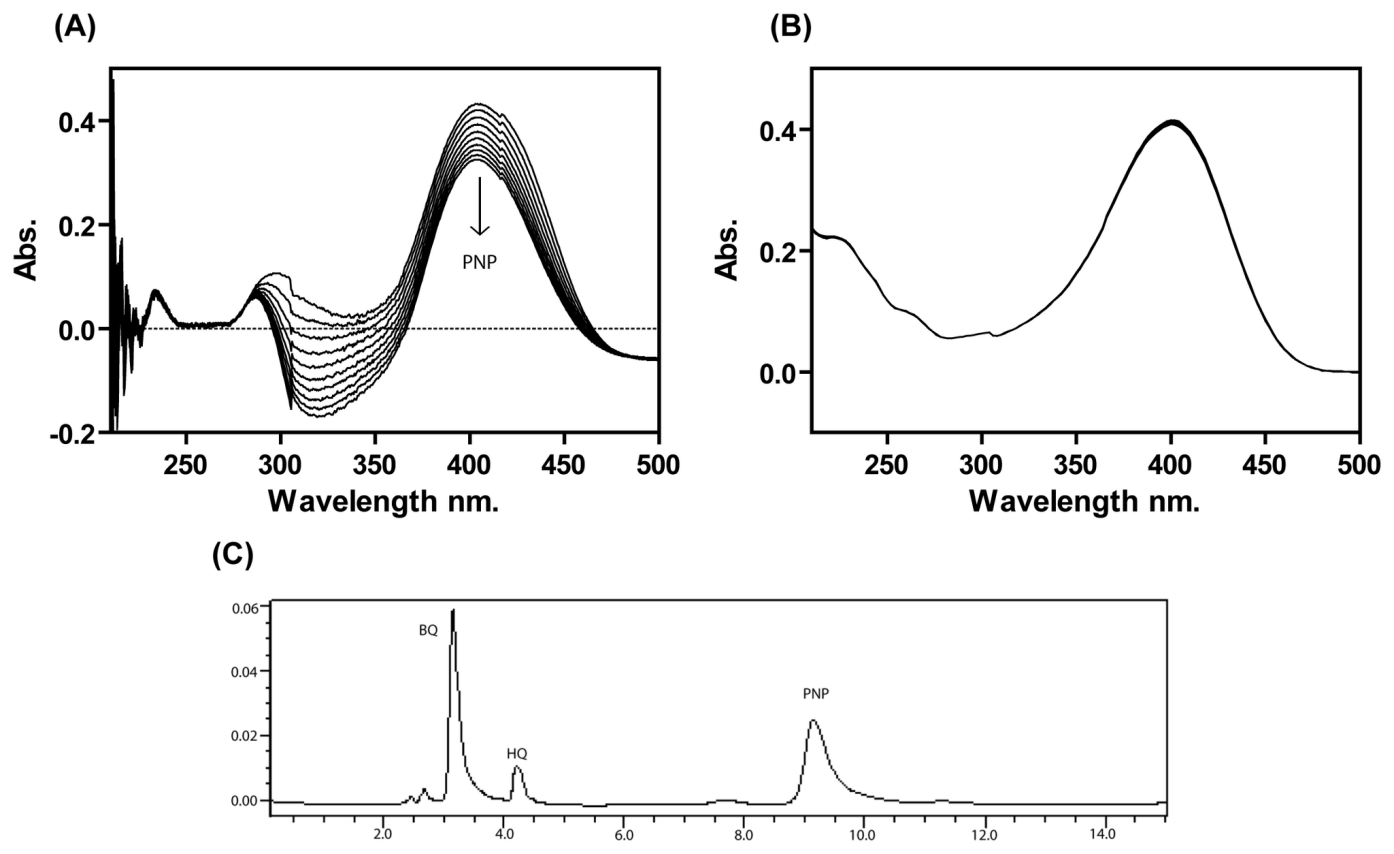
Enzyme assay of *p*-nitrophenol 4-monooxygenase (PnpM) (A) Spectrophotometric analysis PnpM activity as shown by decrease in spectral absorption at 420 and 340 nm corresponding to depletion of *p*-nitrophenol (PNP) and NADH in a time dependent manner. (**B**) Lack of PnpM activity in negative control sample (**C**) Enzymatic transformation of PNP to BQ was confirmed by HPLC analysis.

**Figure 5:**
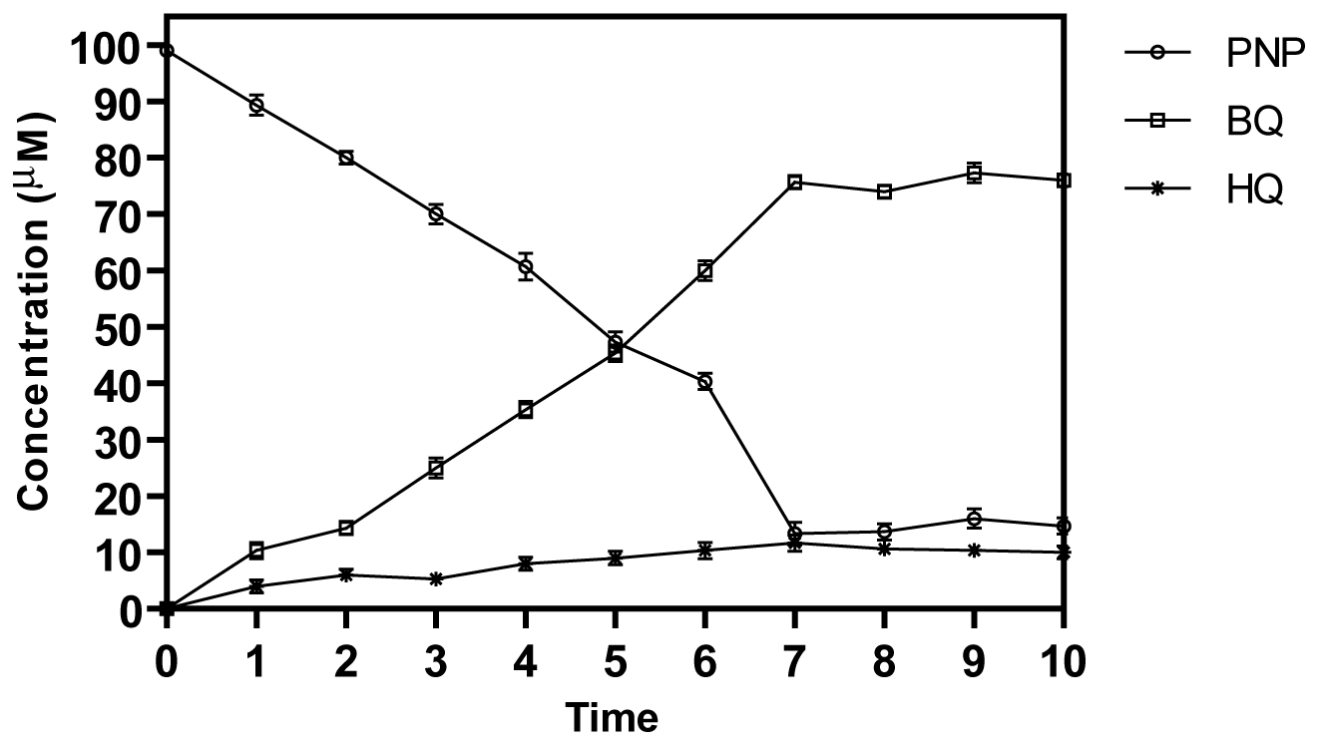
Time course analysis of PNP transformation by purified His-6-PnpA. The samples were collected at different time points and analyzed for the formation of BQ and HQ by HPLC analysis. Standard deviation values were calculated by taking the mean of triplicate experiments.

In *in-vitro* activity assays, a cell extract prepared from recombinant *E. coli* harboring pDEST17-*pnpA* was found positive for the stoichiometric release of NO_2_^-^ ion along with concomitant PNP consumption. Transformation of PNP with stoichiometric NO_2_^-^ ion release was also observed with purified PnpA in the presence of both NADH and NAD(P)H. Effective concentration of NADH required for PnpA was achieved by performing the enzyme activity at different concentrations of NADH (Table 1). In spectrophotometric analyses for monooxygenation, PnpA catalyzed rapid degradation of PNP along with consumption of NADH as indicated by time dependent decrease in the absorbance at 405 nm and 340 nm respectively (Figure 4A). Reference cuvette containing all components of the reaction except purified PnpA did not show any decrease at either 405 nm or 340 nm (Figure 4B). These assays ascertained the identity of PnpA as PnpM. The stoichiometry of PNP consumption in time-course assay was found to be nearly equivalent to the total accumulation of BQ. HPLC analysis of the samples collected from monooxygenation reaction performed with purified PnpA identified BQ as major reaction product by comparison with the authentic standards (Figure 4C). A small amount of HQ was also detected with HPLC analyses (Figure 4C) and this observation could be justified on the basis of abiotic auto-reduction of BQ into HQ in the presence of NADH. Similar non-enzymatic transformation of BQ into HQ has also been reported by Zhang et al., (2009) [25]. Figure 5 shows the time course of PnpA catalyzed transformation of PNP into BQ along with the slight accumulation of hydroquinone in the reaction mixture.

**Table 1:** The kinetic values for the purified His-6-PnpA and His-6-PnpB using Michaelis–Menten kinetics.

**Enzyme**	**Substrate**	**V_max_ (µmole.min^-1^ .nmole^-1^ of protein)**	***K*_*m*_ (µM)**
**PnpA**	*p*-nitrophenol	3.133 ±0.0992	35.37 ± 5.950
	NADH	5.658 ±0.1426	95.99 ± 7.743
**PnpB**	*p*-benzoquinone	1.717 ±0.0464	14.90 ± 2.275
	NAD(P)H	2.952 ±0.0897	118.4 ± 10.96

Kinetic properties of PnpA were determined from Michaelis-Menten curve obtained from spectrophotometric analysis of monooxygenation reaction at different concentrations of PNP with a fixed concentration of NADH and another one obtained with varying concentrations of NADH and fixed concentration of PNP. The V_max_ and *K*_*m*_ values of His-6-PnpA for PNP and NADH were calculated and listed in table 1. Difference in the *K*_*m*_ values of PnpA with reference to PNP and NADH has also been observed with PnpA of *Pseudomonas* sp. WBC-3 (Table S3) [25]. The other noticeable observation was that the purified PnpM of strain SJ98 showed significant differences in *K*_*m*_ and V_max_ values when compared to PnpM of strain WBC-3. Specific activity of PnpM from strain SJ98 is significantly different from those reported for the corresponding purified enzyme from strain WBC-3 (Table S3). 

Purified His-6-PnpA showed a broad temperature optima ranging from 15-30°C with maximum activity at 30°C (Figure S2A). PnpA was found to be functional over the pH range of 6.8- 8.0 with its optimum being the maximum at pH 7.4 (Figure S2B). Since it was observed during the initial screening that the pH optimum for PnpA is at 7.4, therefore, all subsequent biochemical assays carried out for determining the kinetic characteristics were conducted using a buffer with pH of 7.4. In the presence of 10% glycerol, purified His-6-PnpA was stable up-to 7 days at both -20°C and 4°C. The monooxygenase activity of His-6-PnpA was completely abolished in the presence of 100 µM of Cu^2+^, Hg^2+^, and Mo^5+^ salts whereas similar concentrations of Fe^2+^ and Mn^2+^ salts showed no inhibitory effect. 

PnpA was found to catalyze the monooxygenation of different phenolic substrates viz., MNP, ONP, 3M4NP, 4-NC, 2C4NP, 4C3NP, 4C2NP, 2,6DCP, 2,4DNP and 2,5DNP. Of the selected substrates, purified PnpA of strain SJ98 showed the strongest activity with 2C4NP, 4-NC, 2,4-DNP and 3M4NP whereas relatively weaker activity was observed with 2,6-DNP, ONP and PCP (Table 2). A few earlier reports have also suggested for relaxed monooxygenation activity, which resulted in the removal of substituent groups and formation of corresponding quinones from nitro/chloro substituted phenolic compounds in a broad substrate specific manner [25,44].

**Table 2:** Relative percent activity of His-6-PnpA on deferent nitroaromatic compounds.

**Substrate**	**Relative activity (%)**
*p*-Nitrophenol	100 ± 3
o-Nitrophenol	15 ± 2
*m*-Nitrophenol	Nd
3-Methyl 4-nitrophenol	66 ± 3
4-Nitrocatechol	41 ± 2
2-Chloro 4-nitrophenol	25 ± 4
4-Chloro 2-nitrophenol	Nd
2,6-Dichlorophenol	18 ± 2
2,6-Dinitrophenol	15 ± 4
2-Amino 4-nitrophenol	38 ± 3
Pentachlorophenol	12 ± 4
Catechol	Nd
4-Chlorocatechol	Nd

The relative percent activity was measured by HPLC analysis, and standard deviation values were calculated using taking the mean of triplicate experiments.

Nd= activity not observed.

### Biochemical and kinetic properties of purified PnpB

**Figure 6:**
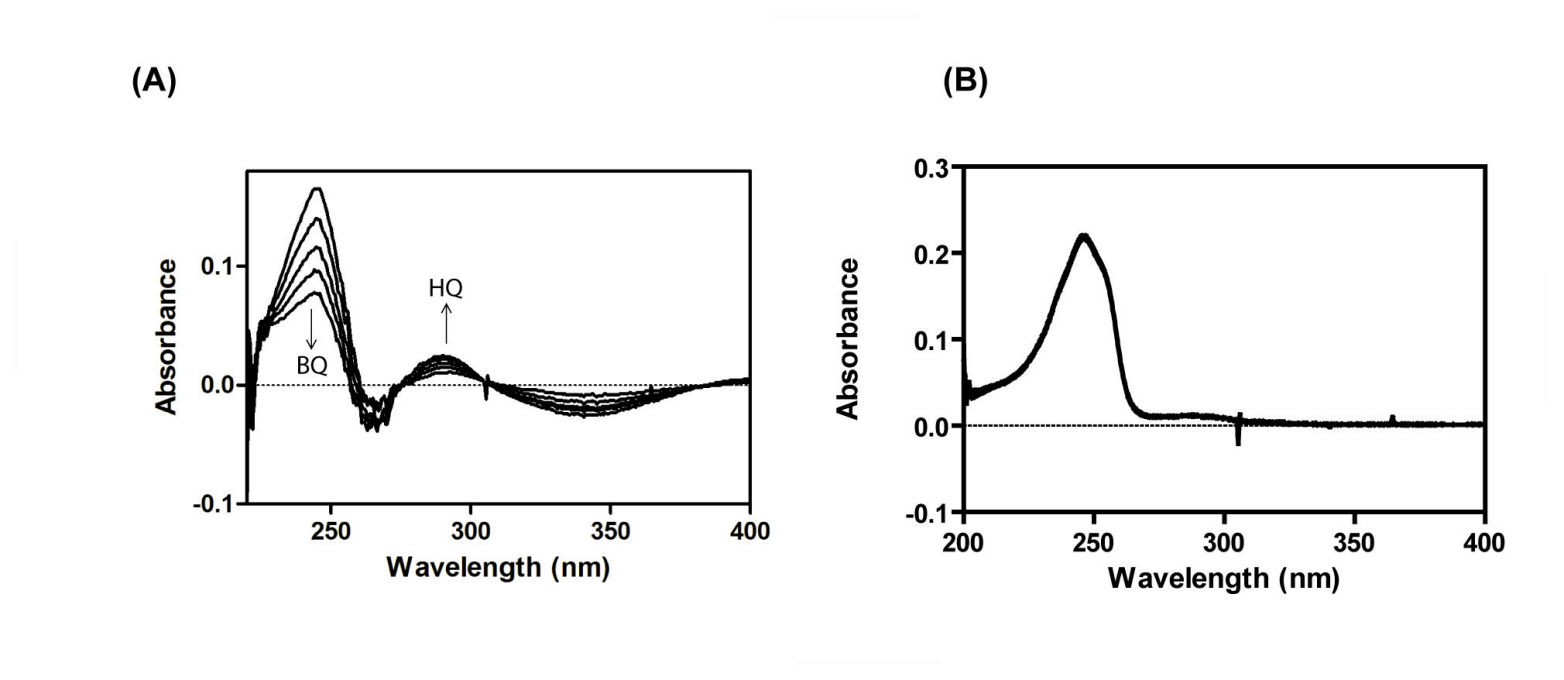
Enzyme assay of p-benzoquinone reductase (BqR) (A) Spectrophotometric analysis of BqR activity of PnpB; depletion in the *p*-benzoquinone along with NAD(P)H (B) Negative control of BqR activity without protein.

The positive BqR activity of purified PnpB was monitored by analyzing the formation of HQ (increased absorbance at 281 nm) and concurrent depletion of BQ (decreased absorbance at 241 nm) in time dependent wavelength scan mode during spectrophotometric analysis (Figure 6A). Similar transformation was not observed in the control reaction mixture lacking His-6-PnpB (Figure 6B). Kinetic properties of PnpB were determined with Michaelis- Menten curves generated with varying concentrations of BQ and NAD(P)H. The V_max_ and *K*_*m*_ of PnpB for BQ and NAD(P)H were calculated and listed in the table 1. It is noticeable that PnpB of strain SJ98 is a single component monomeric protein, whereas the other characterized benzoquinone reductase from strain WBC-3 (as heterologously expressed and purified recombinant protein) is known to exist as dimeric proteins in the functional state [25,41]. At this point, it would be premature and inconclusive to suggest that the difference in the state of oligomerization would also entail to variation in the activity and chemistry of this protein (PnpB). However, it is noteworthy that the difference in the oligomeric state of this protein shows the difference in the kinetic properties of PnpB from strain SJ98 compared to those reported with a dimeric PnpB characterized from *Pseudomonas* sp. WBC-3 [25]. Dendrogram obtained on the basis of multiple sequence alignment of amino-acid sequence of PnpB from strain SJ98 compared to the amino acid sequences of BqR reported from other bacterial strains suggested that PnpB of strain SJ98 constitutes an entirely different clade (Figure S3A & S3B). Further studies of determining the structure-function relation of PnpB would be required to elaborate on its functional evolution. 

Purified His-6-PnpB also showed broad temperature optima ranging from 20 to 40°C (Figure S4A). However, it is active over a relatively narrow range of pH (7.0 to 7.8) with an optimum pH 7.2 (Figure S4B). Therefore, further biochemical characterization of purified PnpB was carried out using a buffer of pH of 7.2. To conclude, the results obtained from the biochemical characterization of the purified PnpA and PnpB ascertained their identity as PnpM and BqR. 

### Phylogenetic analysis of PnpA and PnpB

**Figure 7:**
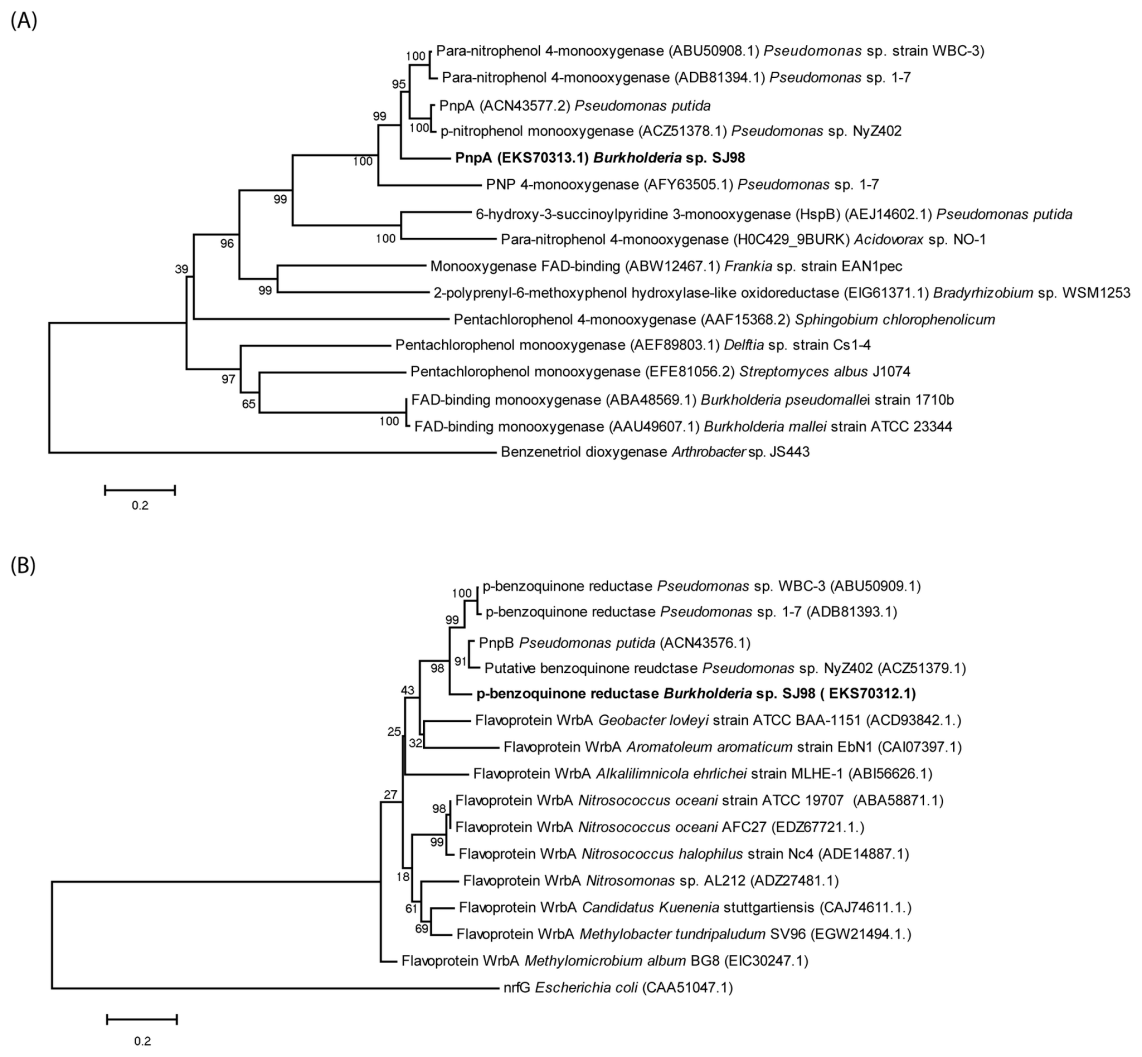
Phylogenetic tree of amino acid sequences (A) *p*-nitrophenol 4-monooxygenase (PnpM), Benzenetriol dioxygenase (BtD) from stain SJ98 was taken as outgroup for the phylogenetic tree for PNP monooxygenases and (B) p-benzoquinone reductase (BqR), nitrite reductase from *E. coli* was taken as an outgroup to create the phylogenetic tree for the benzoquinone reductases. Neighbor joining method was applied at the bootstrap value of 1000. Accession number of the proteins is written in parentheses.

Phylogenetic analysis of PnpA and PnpB of strain SJ98 further substantiated the observation for the presence of *pnpAB* as physically separated gene cluster. In Neighbor Joining phylogenetic tree, PnpA and PnpB from strain SJ98 displayed separate clade from the other PnpMs and BqRs respectively. As shown in Figure 7A, PnpM from strain SJ98 and *Pseudomonas* sp. 1-7 are very different from the PnpMs of other bacterial strains (Figure 7A). Furthermore, PnpB from strain SJ98 also appeared in a different clade (Figure 7B). 

During the recent past, a number of studies have shown identification and characterization of PNP degradation gene clusters from different PNP degrading microorganisms. Identification and characterization of previously unknown gene clusters have helped significantly with the advancements in the field of genome sequencing and annotation. Several studies have reported identification of previously unknown degradation reactions and/or in identification of molecular components (genes, gene clusters) with the important role in previously characterized degradative potentials [45,46]. Furthermore, comparative genomic studies of degradative microorganisms may also help in developing a better understanding about their evolutionary relationships and optimization of bioremediation processes. Significance of the genomic studies is further highlighted in the case of organisms for which genetic tools, e.g. ‘selective gene knockouts’ and ‘complementation studies’ are relatively less well established. Present study conclusively demonstrates the identification of important genes involved in PNP degradation by *Burkholderia* sp. strain SJ98 that had remained elusive with conventional microbial genetic approaches.

## Conclusions

In the present study, we have identified and characterized two ORFs *pnpA* and *pnpB* from the genome sequence of strain SJ98. His-6-PnpA transforms PNP to BQ and has broad substrate specificity while His-6-PnpB transforms BQ to HQ. These genes are physically separated from the other genes (lower pathway genes) involved in the degradation of PNP by strain SJ98. Thus we conclude that genes involved in the degradation of the PNP by strain SJ98 are present in the form of two separate/non-contiguous clusters. This is the first report for identification of such arrangement of PNP degradation gene cluster. We propose that this study would provide valuable insights about underlying molecular mechanism of microbial degradation of PNP and that the strain SJ98 could be used as an important model system for the microbial degradation of PNP.

## Supporting Information

Figure S1 **Determination of molecular weight of PnpB (**A**) Size exclusion chromatography profile of purified functional His-6-PnpB (Rt 11.22 min corresponding to MW ~ 21 kDa).** (**B**) Linear standard curve for the known molecular weight proteins i.e., Feritine (450 kDa), 8.6033 min; Conalbumine, (76.6 kDa) 9.9284 min; Ovalbumine (45 kDa), 10.2454 min; Chymotrypsinogen (25 kDa), 11.1305 min; Ribonuclease A (13.7 kDa) 11.3263 min; Insuline (5.8 kDa) 11.6974 min.
(TIF)

Figure S2 **Relative percent activity of PnpA at different (A) Temperatures and (B) different pH.**
(TIF)

Figure S3 **Multiple sequence alignment (MSA) of (A) PnpB and (B) Dendrogram on the basis of MSA, from *Burkholderia* sp. SJ98 (EKS70312.1), *Pseudomonas* sp. WBC-3 (ABU50909.1), *Pseudomonas putida* (ACN43576.1), *Pseudomonas* sp. 1-7 (ADB81393.1) and *Pseudomonas* sp. NyZ402 (ACZ51379.1).**
(TIF)

Figure S4 **Relative percent activity of PnpB at different (A) Temperatures and (B) different pH.**
(TIF)

Table S1 **Bacterial strains and plasmids used in this study.**
(DOC)

Table S2 **List of primers used in this study.**
(DOC)

Table S3 **Comparison of kinetic properties of *p*-nitrophenol 4-monooxygenase from strain SJ98 with strain WBC-3.**
(DOC)
