# Supplementary figures and images for: Genes Involved in Degradation of para-Nitrophenol Are Differentially Arranged in Form of Non-Contiguous Gene Clusters in Burkholderia sp. strain SJ98
Source: PLoS One. 2013 Dec 23;8(12):e84766. doi: 10.1371/journal.pone.0084766 (PMC3871574; doi:10.1371/journal.pone.0084766)

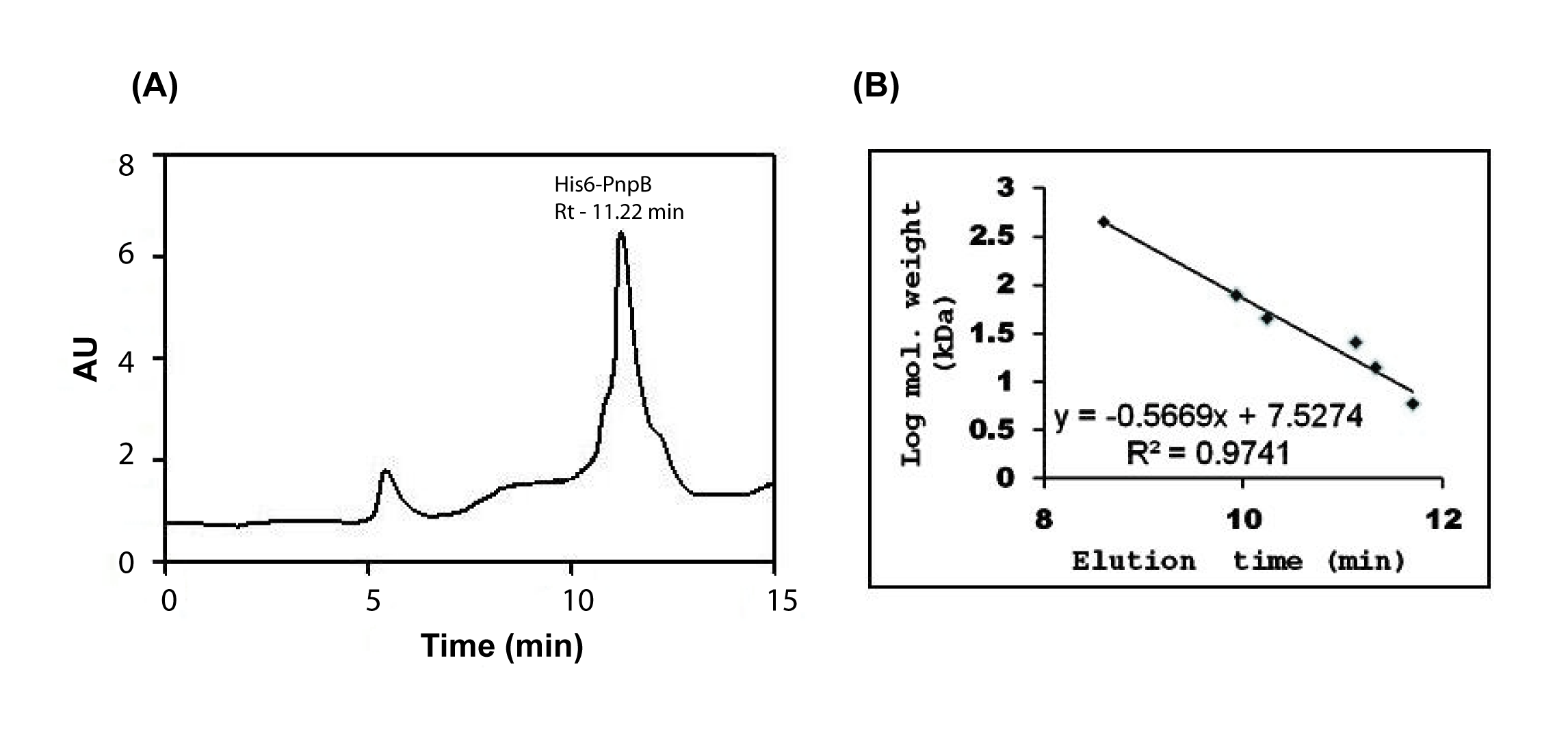

Supplement: Figure S1 — Determination of molecular weight of PnpB (A) Size exclusion chromatography profile of purified functional His-6-PnpB (Rt 11.22 min corresponding to MW ~ 21 kDa). (B) Linear standard curve for the known molecular weight proteins i.e., Feritine (450 kDa), 8.6033 min; Conalbumine, (76.6 kDa) 9.9284 min; Ovalbumine (45 kDa), 10.2454 min; Chymotrypsinogen (25 kDa), 11.1305 min; Ribonuclease A (13.7 kDa) 11.3263 min; Insuline (5.8 kDa) 11.6974 min. (TIF) [file pone.0084766.s001.tif]

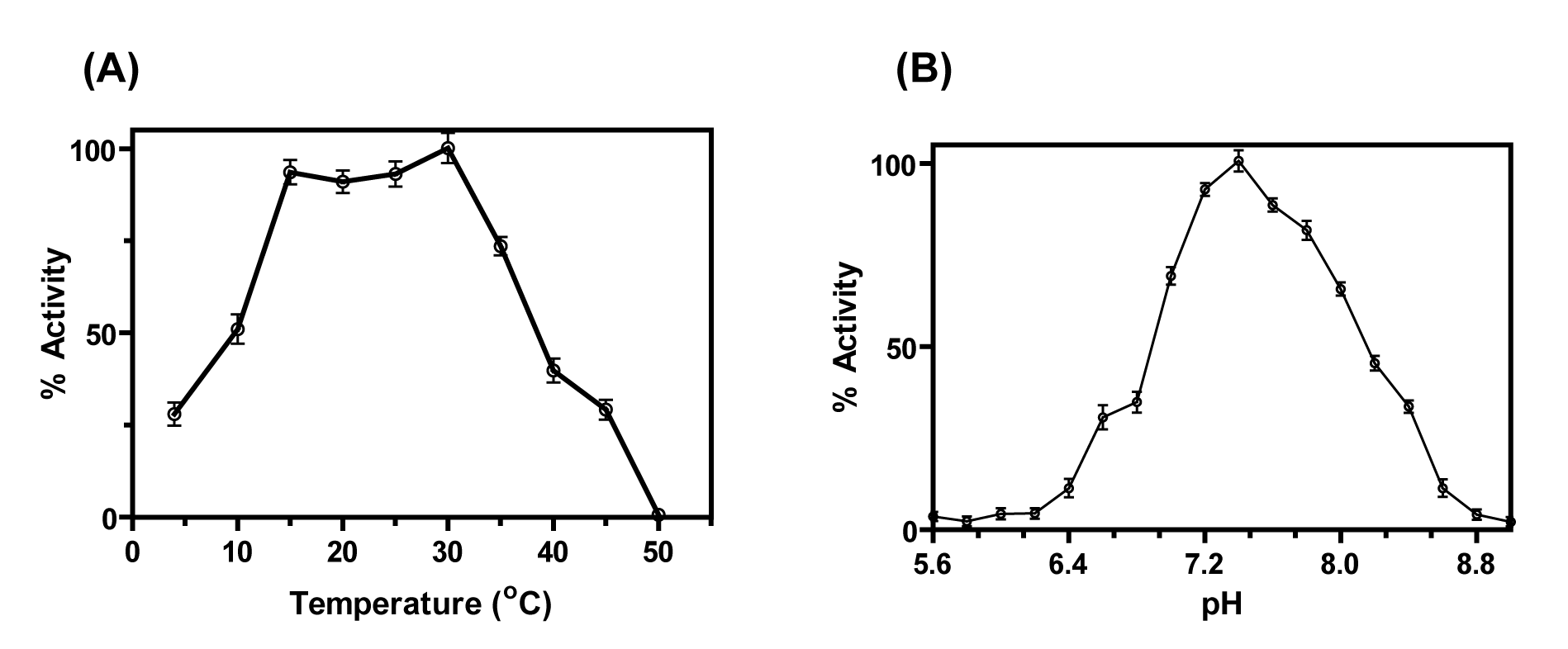

Supplement: Figure S2 — Relative percent activity of PnpA at different (A) Temperatures and (B) different pH. (TIF) [file pone.0084766.s002.tif]

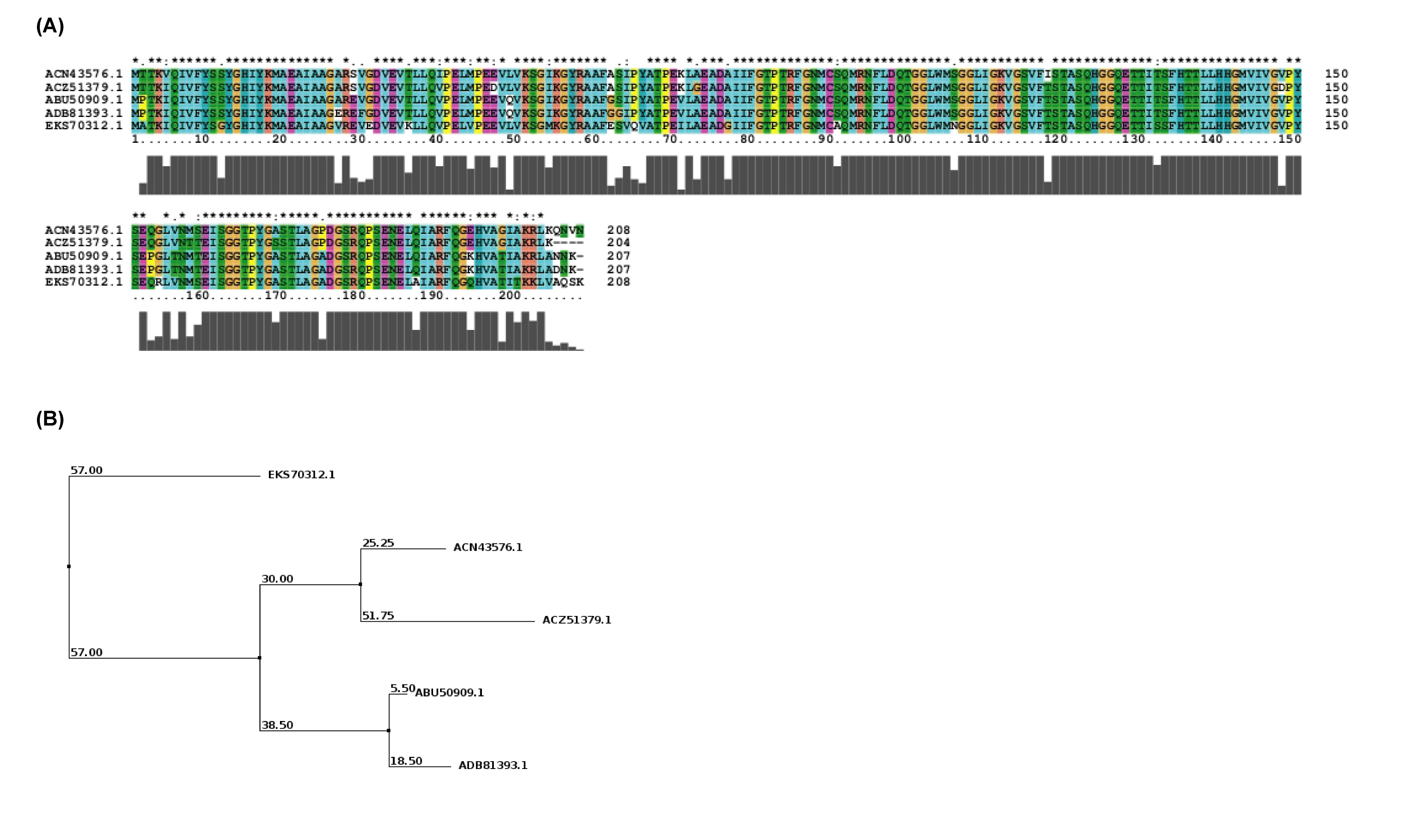

Supplement: Figure S3 — Multiple sequence alignment (MSA) of (A) PnpB and (B) Dendrogram on the basis of MSA, from Burkholderia sp. SJ98 (EKS70312.1), Pseudomonas sp. WBC-3 (ABU50909.1), Pseudomonas putida (ACN43576.1), Pseudomonas sp. 1-7 (ADB81393.1) and Pseudomonas sp. NyZ402 (ACZ51379.1). (TIF) [file pone.0084766.s003.tif]

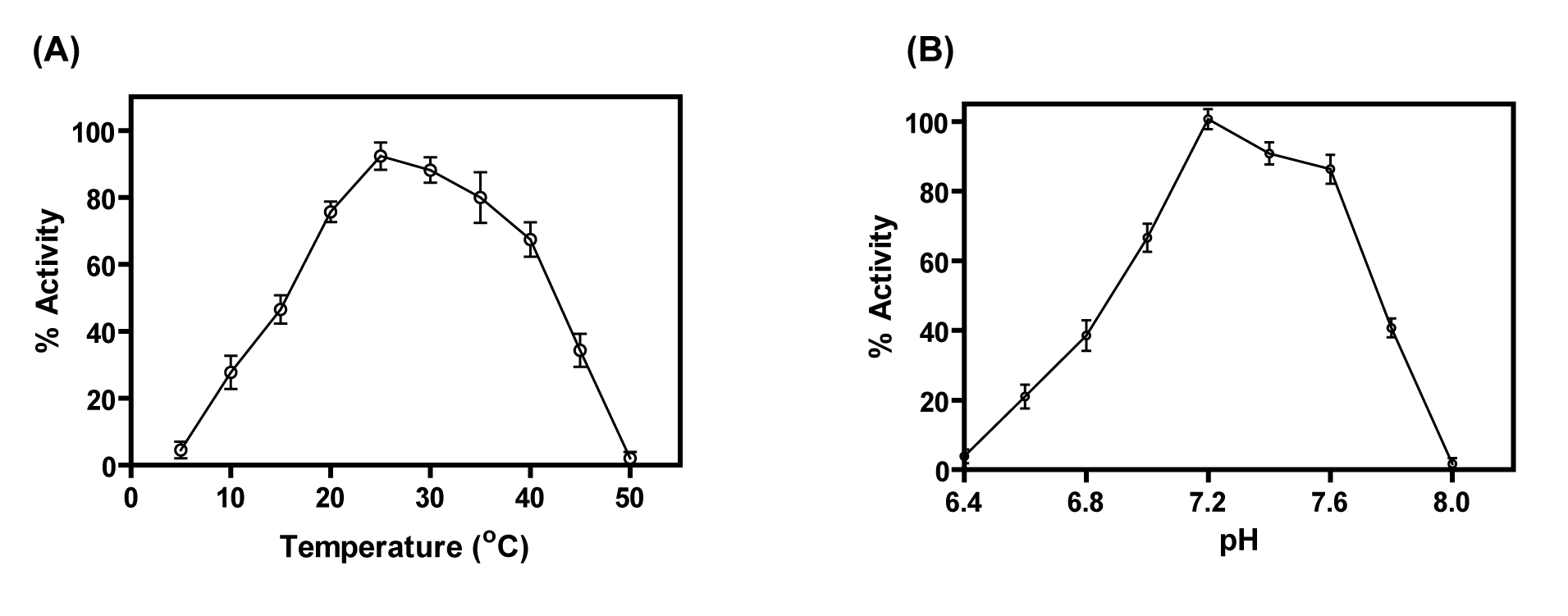

Supplement: Figure S4 — Relative percent activity of PnpB at different (A) Temperatures and (B) different pH. (TIF) [file pone.0084766.s004.tif]
