# Supplementary material for: Genes Involved in Degradation of para-Nitrophenol Are Differentially Arranged in Form of Non-Contiguous Gene Clusters in Burkholderia sp. strain SJ98
Source: PLoS One. 2013 Dec 23;8(12):e84766. doi: 10.1371/journal.pone.0084766 (PMC3871574; doi:10.1371/journal.pone.0084766)
Supplement: Table S1 — Bacterial strains and plasmids used in this study. (DOC) [file pone.0084766.s005.doc]

**Table S1. Bacterial strains and plasmids used in this study**

| **Bacterial strain/ plasmid/ cosmid** | **Characteristic** | **Source or reference** |
| --- | --- | --- |
| **Bacterial strains** |  |  |
| *Burkholderia* sp. strain SJ98 | Wild type PNP degrading isolate | Lab stock and DSM = 23195 |
| *E. coli ­*Top10 | Host strain for cosmid cloning vector  Host strain for GateWay entry clone | Lab stock |
| *E. coli ­*BL-21 AI | Host strain for expression vector (LacY1DE3, F_ompT hsdS, gal, dcm, ara-I) | Invitrogen Inc. CA- USA |
| **Plasmid** |  |  |
| pDONR-221 | Gateway entry cloning vector | Invitrogen Inc. CA, USA |
| pDEST-17 | Gateway expression vector | Invitrogen Inc. CA, USA |
| pDest-*pnpA* | Expression clone with orf *pnpA* of strain SJ98 | This study |
| pDest-*pnpB* | Expression clone with orf *pnpB* of strain SJ98 | This study |
