# Supplementary material for: Genes Involved in Degradation of para-Nitrophenol Are Differentially Arranged in Form of Non-Contiguous Gene Clusters in Burkholderia sp. strain SJ98
Source: PLoS One. 2013 Dec 23;8(12):e84766. doi: 10.1371/journal.pone.0084766 (PMC3871574; doi:10.1371/journal.pone.0084766)
Supplement: Table S2 — List of primers used in this study. (DOC) [file pone.0084766.s006.doc]

**Table S2. List of primers used in this study**

| **Target ORF** | **Primer name** | **Prime sequence (5’ 🡪3’)** | **Application/ purpose** |
| --- | --- | --- | --- |
| **PnpA** | pnpA_F  pnpA_R | GGGGACAAGTTTGTACAAAAAAGCAGGCTTAATGGGCCGACATCTGCAT  GGGGACCACTTTGTACAAGAAAGCTGGGTATTAGAACGCGACCGGATA | PNP monooxygenase |
| **PnpB** | pnpB  pnpB | GGGGACAAGTTTGTACAAAAAAGCAGGCTTAATGGAGACAGACATGCAA  GGGGACCACTTTGTACAAGAAAGCTGGGTATTACTGGATGCAGATGTC | p-benzoquinone reductase |
| **pDONR221 sequencing** | M13_Forward  M13_Reverse | GTAAAACGACGGCCAG  CAGGAAACAGCTATGAC | Sequencing of insert |
| **pDEST17 sequencing** | T7_Forward  T7_Reverse | TAATACGACTCACTATAGGG  GCTAGTTATTGCTCAGCGG | Sequencing of insert |
