# Supplementary material for: Genes Involved in Degradation of para-Nitrophenol Are Differentially Arranged in Form of Non-Contiguous Gene Clusters in Burkholderia sp. strain SJ98
Source: PLoS One. 2013 Dec 23;8(12):e84766. doi: 10.1371/journal.pone.0084766 (PMC3871574; doi:10.1371/journal.pone.0084766)
Supplement: Table S3 — Comparison of kinetic properties of p-nitrophenol 4-monooxygenase from strain SJ98 with strain WBC-3. (DOC) [file pone.0084766.s007.doc]

**Table S3:** Comparison of kinetic properties of *p*-nitrophenol 4-monooxygenase from strain SJ98 with strain WBC-3

|  | | ***Burkholderia* sp. strain SJ98** | | ***Pseudomonas* sp. strain WBC-3** | |
| --- | --- | --- | --- | --- | --- |
| **Enzyme** | **Substrate** | **Vmax (nmole.min^-1^ .µg^-1^ of protein)** | **Km (µM)** | **Specific activity**  **(nmole.min^-1^.ug^-1^)** | **Km (µM)** |
| **PnpA** | PNP | 69.63 ± 2.158 | 35.37 ± 5.950 | 6.9 | 12.0 ±1.6 |
|  | NADH | 123.6 ± 3.101 | 95.99 ± 7.743 | Not given | 137.4 ±12.3 |
